# Modulation of the Hypothalamic Nutrient Sensing Pathways by Sex and Early-Life Stress

**DOI:** 10.3389/fnins.2021.695367

**Published:** 2021-07-23

**Authors:** Silvie R. Ruigrok, Nina Stöberl, Kit-Yi Yam, Chiara de Lucia, Paul J. Lucassen, Sandrine Thuret, Aniko Korosi

**Affiliations:** ^1^Center for Neuroscience, Swammerdam Institute for Life Sciences, University of Amsterdam, Amsterdam, Netherlands; ^2^Department of Basic and Clinical Neuroscience, Institute of Psychiatry, Psychology and Neuroscience, King’s College London, London, United Kingdom

**Keywords:** nutrient sensing, hypothalamus, sex differences, early-life stress, metabolism

## Abstract

There are sex differences in metabolic disease risk, and early-life stress (ES) increases the risk to develop such diseases, potentially in a sex-specific manner. It remains to be understood, however, how sex and ES affect such metabolic vulnerability. The hypothalamus regulates food intake and energy expenditure by sensing the organism’s energy state via metabolic hormones (leptin, insulin, ghrelin) and nutrients (glucose, fatty acids). Here, we investigated if and how sex and ES alter hypothalamic nutrient sensing short and long-term. ES was induced in mice by limiting the bedding and nesting material from postnatal day (P)2-P9, and the expression of genes critical for hypothalamic nutrient sensing were studied in male and female offspring, both at P9 and in adulthood (P180). At P9, we observed a sex difference in both *Ppargc1a* and *Lepr* expression, while the latter was also increased in ES-exposed animals relative to controls. In adulthood, we found sex differences in *Acacb*, *Agrp*, and *Npy* expression, whereas ES did not affect the expression of genes involved in hypothalamic nutrient sensing. Thus, we observe a pervasive sex difference in nutrient sensing pathways and a targeted modulation of this pathway by ES early in life. Future research is needed to address if the modulation of these pathways by sex and ES is involved in the differential vulnerability to metabolic diseases.

## Introduction

The prevalence of obesity and the related chronic diseases rise, and postulate a global health concern (Malik et al., 2013). While both men and women are affected, there is a differential risk to develop metabolic diseases between sexes (Kanter and Caballero, 2012; Kautzky-Willer et al., 2016). Worldwide, more women than men are obese, although the prevalence of overweight and obesity among men and women vastly fluctuates between countries (Kanter and Caballero, 2012). Under healthy bodyweight conditions, men and women differ in fat quantity and distribution (Power and Schulkin, 2008; Fuente-Martín et al., 2013), and rodent studies show for example that males and females differently respond to high-fat diets (Grove et al., 2010; Estrany et al., 2011, 2013; Garg et al., 2011).

Next to sex as determinant of metabolic vulnerability, there is also evidence that the perinatal environment is critical for establishing a metabolic set point defining later-life metabolic health (Bouret and Simerly, 2006; Levin, 2006; Bouret, 2009; Yam et al., 2015, 2017a). Exposure to stress during this sensitive developmental period (e.g., neglect or abuse) increases the risk to develop metabolic diseases, including obesity and diabetes, later in life (Alciati et al., 2013; Danese and Tan, 2014; Basu et al., 2017). Indeed, we have previously shown in mice that early-life stress (ES)-exposed males and females have a leaner phenotype when fed a (healthy) standard chow diet, but that ES-exposed males accumulated more fat when later fed a western-style diet (WSD) with a moderate fat content, while how females respond to such a diet was not investigated in this study (Yam et al., 2017a). There is in fact increasing evidence that the effects of ES may be sexually dimorphic (Boynton-Jarrett et al., 2010; Kozyrskyj et al., 2011; Murphy et al., 2018; Park et al., 2018). However, the mechanisms contributing to the sex and ES-induced impacts on metabolic regulation are not well understood.

The arcuate nucleus of the hypothalamus (ARH) regulates energy homeostasis by controlling food intake, energy expenditure and glucose metabolism, and it has been proposed that its development is important for the establishment of the metabolic setpoint (Bouret and Simerly, 2006; Bouret, 2009, 2012; Dearden and Ozanne, 2015; Ramamoorthy et al., 2015). The ARH integrates a wide range of inputs, including those from metabolic hormones and peripheral nutrients (Lam et al., 2005; Timper and Brüning, 2017). The key neuropeptides regulating food intake in the ARH are agouti-related protein (AgRP) and neuropeptide Y (NPY), which stimulate food intake while inhibiting energy expenditure, and proopiomelanocortin (POMC), which has opposite regulatory effects (Barsh and Schwartz, 2002; Timper and Brüning, 2017). Metabolic hormones, including leptin, ghrelin and insulin, act on ARH neurons by binding to their respective receptors. Leptin is an adipokine secreted by the white adipose tissue, which, via binding to its receptor (LepR) inhibits food intake (Friedman, 2014). Insulin, via binding to the insulin receptor (InsR), has a comparable effect as leptin on food intake (Porte and Woods, 1981; Könner et al., 2009), whereas ghrelin (primarily secreted by the stomach) binds to the growth hormone secretagogue receptor (GHSR) (Gil-Campos et al., 2006), and stimulates food intake and bodyweight gain (Wren et al., 2001). Importantly, we have shown before that ES reduced circulating leptin levels and leptin expression in the adipose tissue, while increasing *Lepr* expression in the choroid plexus, which is an important side of leptin entry into the brain (Zlokovic et al., 2000), in both males and females when fed standard chow diet (Yam et al., 2017a). ES furthermore affected circulating ghrelin levels in an age-dependent manner specifically in females, while altering the fiber density of NPY and AgRP in both males and females at postnatal day (P)14, i.e., when the hypothalamus is fully developed (Yam et al., 2017b).

As mentioned above, next to the hormonal regulation of food intake, circulating nutrients [e.g., glucose, fatty acids (FA)] are sensed in the hypothalamus and provide information about the energy state (Matzinger et al., 2000; Lam et al., 2005; Migrenne et al., 2006; Rijnsburger et al., 2016; Le Foll, 2019). More specifically, FA metabolism in the hypothalamus is suggested to function as sensor for nutrient/energy availability integrating both nutritional and hormonal signals. The intracellular pool of long-chain FAs depends on both biosynthetic and oxidative pathways (breakdown of FAs) (Lam et al., 2005) and thought to signal energy abundance (Lam et al., 2005; Dragano et al., 2020). Indeed, FA administration has been shown to suppress *Npy* and *Agrp* expression *in vitro* (Ma et al., 2017), and *in vivo* experiments showed that intracerebroventricular FA administration inhibits food intake and *Npy* expression (Obici et al., 2002). Furthermore, manipulating key proteins involved in both FA synthesis and oxidation (e.g., FASN, CPT1, and ACC) centrally affects food intake and bodyweight (Loftus et al., 2000; Shimokawa et al., 2002; Obici et al., 2003; Cesquini et al., 2008; Jo et al., 2009), and central peroxisome proliferator–activated receptors (PPARs), a family of transcription factors regulating lipid and glucose metabolism, have also been shown to modulate food intake and fat mass (Lu et al., 2011; Ryan et al., 2011; Kocalis et al., 2012). Importantly, glucose, leptin, ghrelin and insulin also affect FA metabolism pathways in the hypothalamus (Lam et al., 2005; Gao et al., 2007; López et al., 2008; Lage et al., 2010; Diéguez et al., 2011; Taïb et al., 2013). Thus, hypothalamic FA metabolism integrates nutrient and hormonal signals and affects hypothalamic neuropeptide expression and food intake.

Yet, it remains to be determined if and how sex and ES impact hypothalamic neuropeptide systems and nutrient sensing pathways. Therefore, we here investigated the gene expression of hypothalamic neuropeptides, hormone receptors and fatty acid metabolism at P9 and P180 in male and female control (CTL) and ES-exposed mice. We studied this in the cohort of mice that we used to report on the metabolic effects of ES in males and females (Yam et al., 2017a), giving us the opportunity to link these gene expression profiles to their metabolic phenotype. We show pervasive sex differences in nutrient sensing pathways and, early in life, a targeted modulation of these pathways by ES.

## Materials and Methods

### Animals and Breeding

In total 17 male and 14 female pups, and 17 male and 13 female adult mice were used for this study. Animals were kept under standard housing conditions (temperature 20–22°C, 40–60% humidity, 12/12h light/dark schedule), and fed standard chow (CRM (P), 801722, Standard Diets Services, Essex, United Kingdom, 3.585 kcal/g, with 22% protein, 9% fat, and 69% carbohydrates) and water provided *ad libitum*. All experimental procedures were conducted under national law and European Union directives on animal experiments, and were approved by the animal welfare committee of the University of Amsterdam.

To standardize the perinatal environment, experimental animals were bred in house. For breeding, 8-week-old C57Bl/6J female and male mice were purchased from Envigo Laboratories B.V. (Venray, Netherlands), and allowed to habituate for 1–2 weeks. Next, two primiparous females were housed with one adult male to allow for mating. After 1 week, the male was removed and females were housed together for another week in a clean cage with nest material (square piece of cotton) to practice, and after another week, females were housed individually in a type II cage with nesting material and filtertop. Starting from 18 days after the start of the breeding, dams were checked for the birth of pups each morning before 09:00 a.m. When a litter was born, the previous day was designated as postnatal day 0.

### ES Paradigm

The limited nesting and bedding material model was used to induce ES from P2 to P9, as described previously (Rice et al., 2008; Naninck et al., 2015). In short, litters were culled to 6 pups, including both males and females, and randomly allocated to the control (CTL) or ES condition at P2. Litters with less than 5 pups were excluded. CTL cages had a standard amount of sawdust and one square piece of cotton nesting material (5 × 5 cm, Tecnilab-BMI, Someren, The Netherlands), whereas ES cages had a thin layer of sawdust on the bottom, covered with a fine-gauge stainless steel mesh 1 cm above the bottom of the cage, and half a square piece of cotton nesting material (2.5 × 5 cm). All cages were covered with a filtertop. For the adult experiment, litters were moved to new cages containing standard amounts of sawdust at P9, and left undisturbed until weaning at P21, after which they were group-housed with same sex littermates.

### Tissue Preparation

To study the direct effects of ES on nutrient sensing pathways in male and female offspring, pups were sacrificed in the morning of P9 by rapid decapitation. For adult studies, animals were fasted for 4 hours and sacrificed by rapid decapitation at P180. The hypothalamus was rapidly dissected, snap frozen and stored at −80°C. RNA was obtained using Trizol (Invitrogen, Carlsbad, CA, United States) according to manufacturer’s instructions and stored at −80°C until further use. Reverse transcription of RNA into cDNA was done with SuperScript II Reverse Transcriptase (Invitrogen, Carlsbad, CA, United States), and cDNA was stored at −20°C until further analysis.

### Real-Time PCR

Relative gene expression was measured using a 7500 Real-time PCR system (Applied Biosystems, Foster City, CA, United States). In short, for all genes with exception of the leptin receptor (see below), hot FirePol EvaGreen Mastermix (Solis Biodyne, Tartu, Estonia) was used. Primers (Eurogentec, Liege, Belgium) of target and reference genes all had an efficiency between 90 and 110% (Supplementary Table 1). Gene specific forward and reverse primers together with cDNA template were added to the reaction mix according to manufacturer’s instructions. Cycling conditions were as follows: 15 min polymerase activation at 95°C, followed by 40 cycles of replication (15 s at 95°C, 20 s at 65°C, and 35 s at 72°C) and a dissociation program. For the quantification of the leptin receptor, Taqman^®^ probes were used. TaqMan^®^ Gene Expression Master Mix (4369016, Thermo Fisher Scientific, Waltham, MA, United States) was added to cDNA template and *Lepr* Taqman^®^ probes (6749720_1, Thermo Fisher Scientific, Waltham, MA, United States), and the following cycling conditions were used: 2 min 50°C and 10 min 95°C for activation, followed by 40 cycles of replication (15 s at 95°C, 1 min at 60°C) and a dissociation program. Gene expression was calculated with the ΔΔCt method in Qbase+ (Biogazelle, Zwijnaarde, Belgium) and normalized for two reference genes (*Rpl13a* and *Rplp0* for P9, *Tbp* and *Tuba1a* for P180) which were tested for stability.

### Statistical Analysis

Data were analyzed with SPSS 25.0 (IBM software, Armonk, NY, United States), R studio 1.2.1335 (R Core Team, 2018) and Graphpad Prism 6 (Graphpad software, San Diego, CA, United States). All data are presented as mean ± standard error of the mean (SEM), and when *p* < 0.05, data was considered statistically significant. Gene expression data was scaled against the CTL male group, and log transformed for statistical analysis. Outliers were identified in SPSS and removed, and data was analyzed with a 2-way ANOVA with condition and sex as predictor variables. Multiple mice from the same litter were included in this study, and therefore data are nested. We verified if litter contributed to the outcome by performing mixed model analysis with litter as random factor. Litter did not affect any of the outcome variables. In addition, to test whether estrous cycle contributed to the variable outcomes, we performed mixed model with estrous cycle as random factor. Estrous cycle did not affect any of the outcomes. Total white adipose tissue levels (as percentage of bodyweight) and circulating leptin levels (previously published in Yam et al., 2017a) were correlated to hypothalamic gene expression levels with R studio software (version 1.2.1335) (R Core Team, 2018). Pearson correlations were calculated based on complete pairwise cases, and correlation coefficients were tested against critical values on a two-tailed distribution (alpha = 0.05).

## Results

### Effects of ES on Hypothalamic Gene Expression in Male and Female Offspring at P9

**FIGURE 1:**
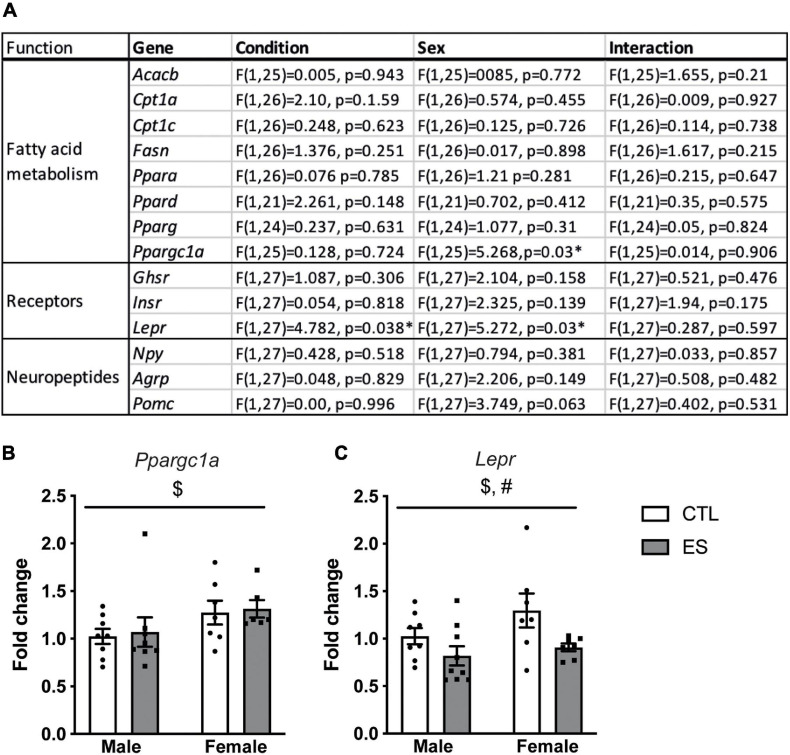
**(A)** Effects of ES and sex on hypothalamic gene expression at P9. **(B)** Hypothalamic *Ppargc1a* expression was higher in females. **(C)** *Lepr* expression was higher in females and lower in ES-exposed animals. Indicated is mean ± SEM, *p* < 0.05. # Main effect of condition; * = significant effect; $ main effect of sex.

Figure 1A shows a full overview of the effects of ES and sex on hypothalamic gene expression. Among the genes that regulate fatty acid metabolism, *Ppargc1a* was higher in females compared to males (Figure 1B). Moreover, *Lepr* expression was higher in females, and lower in ES-exposed males and females compared to their respective controls (Figure 1C). The expression of other receptors and hypothalamic neuropeptides were not affected by sex or ES.

### Effects of ES on Hypothalamic Gene Expression in Male and Female Mice at P180

**FIGURE 2:**
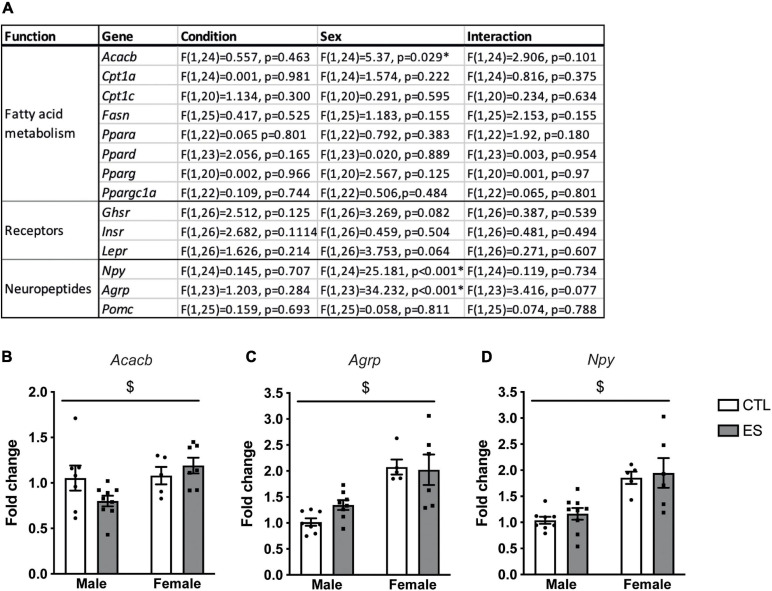
**(A)** Effects of ES and sex on hypothalamic gene expression in adulthood. **(B)** *Acacb* expression was higher in females. **(C)** Expression of *Agrp* was higher in females. **(D)** *Npy* expression was higher in females. Indicated is mean ± SEM, *p* < 0.05. * = significant effect; $ main effect of sex.

Figure 2A shows an overview of the effects of ES and sex on hypothalamic gene expression in adulthood (P180). *Acacb* expression was higher in females (Figure 2B). Moreover *Agrp* (Figure 2C) and *Npy* (Figure 2D) expression were higher in females compared to males. There were no effects of ES on the hypothalamic expression of hormone receptors, genes in fatty acid metabolism, or neuropeptides in adulthood.

### Hypothalamic Gene Expression Correlates With Metabolic Readouts in Adulthood

**FIGURE 3:**
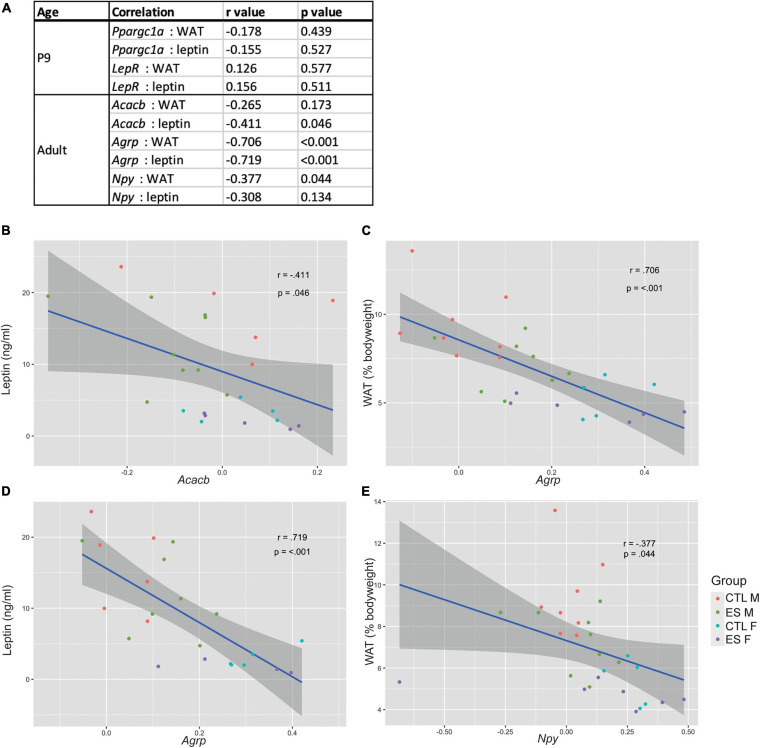
**(A)** Correlations between significantly affected genes and metabolic readouts at P9 and in adulthood. **(B)** *Acacb* expression correlated with leptin levels. **(C)** *Agrp* expression correlated with white adipose tissue (WAT) levels. **(D)** *Agrp* expression correlated with leptin levels. **(E)** *Npy* expression correlated with WAT levels.

In the same cohort of mice as used in the current study, we previously reported that ES decreased white adipose tissue (WAT) levels at P9 and in adulthood, as well as reduced leptin expression and circulating leptin levels in both males and females (see Yam et al., 2017a). To explore whether hypothalamic gene expression is related to the metabolic profile, we correlated the genes that were significantly affected by either ES or sex with white adipose tissue (WAT) levels and circulating leptin levels (Figure 3A). At P9, *Ppargc1a* and *LepR* expression did not correlate with either WAT or leptin levels. In adulthood, *Acacb* expression correlated with leptin levels (Figure 3B), *Agrp* expression correlated with both WAT and leptin levels (Figures 3C,D), and *Npy* expression correlated with WAT levels (Figure 3E).

## Discussion

We presented here for the first time the effect of sex and ES on hypothalamic fatty acid metabolism, hormone receptors and neuropeptides at P9 and in adulthood. Pervasive sex differences emerge at both ages with a targeted impact of ES at P9, which was independent of sex. In particular, at P9, females had higher *Ppargc1a* expression compared to males. Moreover, while *Lepr* expression was higher in females compared to males, ES decreased *Lepr* expression independent of sex. In adulthood, females, compared to males, had higher *Acacb*, *Npy*, and *Agrp* expression. We will first discuss the sex differences in hypothalamic gene expression at P9 and adulthood, followed by the effects of ES, and will relate this to the previously described metabolic phenotype (Yam et al., 2017a,b).

### Sex Differences in Hypothalamic Nutrient Sensing

At P9, females had higher *Ppargc1a* and *Lepr* expression compared to males. At this age, females also have a lower bodyweight compared to males, whereas their adiposity (as percentage of bodyweight) is similar (Yam et al., 2017a). PGC-1α (*Ppargc1a)* is known as transcription factor that responds to environmental and energy signals and targets the PPAR family (Sugden et al., 2010). As such, it is involved in the regulation of mitochondrial oxidative metabolism, glucose and lipid homeostasis, and was implicated in metabolic diseases (Lin et al., 2005). Whole body PGC-1α null mice are lean, resistant to diet-induced obesity and show hyperactivity (Lin et al., 2004), and also a selective inactivation of hypothalamic PGC-1α protected mice against diet-induced obesity (Ma et al., 2010). A high-fat diet furthermore downregulated hypothalamic PGC-1α in male but not female mice (Morselli et al., 2014). Since we did not find significant correlations between *Ppargc1a* expression and adiposity or leptin levels, it remains to be determined what is the functional implication of the observed sex differences in *Ppargc1a* expression early in life.

The increased *Lepr* expression in females at P9, despite similar leptin levels in male and female pups (Yam et al., 2017a), suggests there are sex-differences in leptin signaling in the hypothalamus. Sex differences in *Lepr* expression seem to be brain region specific, as hippocampal *LepR* expression was similar in males and females (Yam et al., 2017a). Leptin signaling early in life, rather than modulating food intake, is crucial for the development of the hypothalamic circuitry (Ahima et al., 1998; Mistry et al., 1999; Bouret et al., 2004; Granado et al., 2012). Although in the current study we did not observe sex differences in whole hypothalamic neuropeptide expression levels at this age, with a more anatomically precise approach we previously described higher ARH NPY fiber density in females compared to males at P14 (Yam et al., 2017b).

In adulthood, females had higher *Acacb*, *Agrp*, and *Npy* levels in the hypothalamus compared to males. Female mice at this age weigh less, and have lower adiposity independent of bodyweight, as well as lower circulating leptin levels when fed a standard chow diet (Yam et al., 2017a). Acetyl-CoA carboxylases (ACCs) occur in two isozymes [ACC1 (*Acaca*) and ACC2 (*Acacb*)] that are activated in the fed state. While ACC1 plays a key role in fatty acid synthases, ACC2 inhibits β-oxidation (Tong and Harwood, 2006), and higher *Acacb* expression thus indicates more inhibition of β-oxidation. The higher hypothalamic *Acacb* expression in female hypothalamus is in line with a previous report showing higher hepatic *Acacb* levels in female mice (Tuazon et al., 2015). To get a better understanding of what could be the functional implication of such sex-specific change in expression, is notable that polymorphisms in the *Acacb* gene influence the risk on developing metabolic syndrome in humans (Phillips et al., 2010). Whether ACCs are involved in sex differences in metabolism remains to be determined.

Increased *Acacb* expression in females suggests lower *Npy* and *Agrp* gene expression (Rijnsburger et al., 2016). In contrast, we observed higher hypothalamic expression of *Npy* and *Agrp* mRNA in females compared to males. A previous study found lower numbers of NPY expressing cells in the ARH of females (in proestrus) compared to males (Urban et al., 1993), and for *Agrp*, both lower (Viveros et al., 2010) as well as higher (Lensing et al., 2016) expression levels have been described in females. In fact, *Npy* and *Agrp* expression are affected by sex steroids. Testosterone increases *Npy* expression and release (Sahu et al., 1989; Urban et al., 1993), and in females, *Npy* and *Agrp* expression fluctuates throughout the estrous cycle, parallel to the cyclic changes in food intake and bodyweight (Olofsson et al., 2009; Fontana et al., 2014). Although estrous cycle was not a contributing factor in our statistical models, our study was not designed to specifically investigate the effect of estrous cycle in females. Another difference between our study and others is whether the animals were fasted before sacrificing. We fasted the mice for 4 h, while other studies describe no fasting period (Urban et al., 1993; Viveros et al., 2010; Lensing et al., 2016). Indeed, female rats show an increase in food intake after fasting, while male rats do not show such rebound feeding (Funabashi et al., 2009). This was accompanied by an increased number of activated hypothalamic orexin neurons in females (independent of estrous cycle) but not males. Sex differences in *Agrp* and *Npy* expression dynamics thus require further investigation.

In our study, despite increased *Npy* and *Agrp* expression in females, which stimulate food intake and reduce energy expenditure (Timper and Brüning, 2017), females were leaner and did not eat more of the standard chow than males (food intake after fasting was not investigated) (Yam et al., 2017a). In fact, male rodents are more vulnerable to develop adiposity and have more greatly impaired glucose tolerance upon long-term high-fat diet feeding (Grove et al., 2010; Estrany et al., 2011, 2013). Sex differences in the quantity and functioning of fat depots (Fuente-Martín et al., 2013) likely contribute to this increased metabolic vulnerability in males, and the same applies to the sympathetic innervation and projections from the hypothalamus to the adipose tissue (Adler et al., 2012). We here show that *Acacb*, *Agrp*, and *Npy*, genes that were differentially expressed between males and females, correlated with metabolic readouts (adiposity and/or leptin levels). Although these correlation analyses cannot imply causality nor directionality, our data suggest that sex-differences in nutrient sensing pathways could also contribute to this sex-bias in metabolic vulnerability, an avenue worth investigating in the future.

### Effects of ES on Nutrient Sensing Pathways

ES exposure affected hypothalamic nutrient sensing pathways early in life, in a targeted manner. More specifically, at P9, *Lepr* expression was lower in ES-exposed mice. At this age, ES also led to a reduction in circulating leptin levels, that was notably similar in males and females, and did not affect hippocampal *Lepr* expression (Yam et al., 2017a), indicative of brain-region specific effects of ES on *Lepr* expression. Moreover, the reduction in leptin levels together with the lower hypothalamic *Lepr* expression suggests reductions in leptin signaling during hypothalamic development. In line with the altered hypothalamic leptin signaling at P9, we had previously found that at P14, ES also affected NPY and AgRP fiber density in the ARH and paraventricular nucleus, respectively (Yam et al., 2017b). In our current study, however, ES did not affect the gene expression *Npy* and *Agrp* in whole hypothalamic lysates. This could be due to the different method of assessment (i.e., gene expression of the whole hypothalamus versus fiber density in specific regions), and/or depend on the specific developmental stage due to the rapid development of the hypothalamus during the first two postnatal weeks (Bouret and Simerly, 2006; Bouret, 2012). Despite ES-induced changes in ghrelin levels in females at P9 (Yam et al., 2017b), hypothalamic *Ghsr* levels were not affected by ES, nor did ES affect the expression of genes involved in fatty acid metabolism. Thus, ES alters the leptin system at P9, whereas hypothalamic fatty acid metabolism seems unaffected. Interestingly, the leptin signaling pathway has been suggested to be a top upstream regulator of the effects of chronic stress in the prefrontal cortex, indicating leptin could be one of the key mediators by which stress exerts its effect on the brain (Musaelyan et al., 2020). Whether this might be the case also in the context of ES remains to be addressed.

In adulthood, ES did no longer impact the expression of genes involved in hypothalamic nutrient sensing. While we and others have previously shown lasting effects of ES on adipose tissue levels under basal circumstances and/or in the response to unhealthy diets (Murphy et al., 2017, 2018; Yam et al., 2017a), it remains unclear what leads to these ES-induced metabolic alterations. Investigating the expression of genes involved in nutrient sensing in (whole) hypothalamic samples provides a first indication of potential effects of ES on these pathways. However, it would be important in future studies to investigate these pathways with better spatial resolution and on the protein level, and next to under basal circumstances, also in response to an unhealthy diet. For example, we have previously shown that ES alters hypothalamic microglia in response to a western-style diet, but not under standard dietary circumstances (Ruigrok et al., 2021). Next to the hypothalamus, also other brain circuits and/or peripheral organs could be involved in the ES-induced metabolic risk. For example, we have previously shown ES-induced changes in the adipose tissue gene expression (Yam et al., 2017a; Ruigrok et al., 2021). Adipose tissue has a crucial role in regulating energy homeostasis, and its dysfunction has been related to the negative health consequences associated with obesity (Longo et al., 2019). In addition, the microbiome is implicated in obesity development (Davis, 2016), and has been shown to be affected by ES exposure (O’Mahony et al., 2009). ES has furthermore been shown to affect the reward circuitry (Spyrka et al., 2020), which is involved in the hedonic aspects of food intake, an important aspect of human obesity development, and there is initial evidence that food preference indeed is affected by ES exposure (Lussana et al., 2008; Jackson and Vaughn, 2019). In addition to the above described sex differences in the hypothalamus and adipose tissue, sex differences in the microbiome and reward circuitry have also been reported (Fuente-Martín et al., 2013; Becker and Chartoff, 2019; Kim et al., 2020), but it remains to be determined whether ES affects these systems in a sex-dependent manner. More research is thus needed to further elucidate how ES increases the risk for developing obesity, potentially in a sex-specific manner.

## Conclusion

To the best of our knowledge, this is the first description of effects of ES and sex on hypothalamic nutrient sensing pathways. Clear sex differences were present in these pathways, while ES induced a minor modulation early in life that was similar in both sexes. Our data contribute to a further understanding of sex differences in the circuits that regulate metabolism and the impact of ES on these pathways.

## Data Availability Statement

The raw data supporting the conclusions of this article will be made available by the authors, without undue reservation.

## Ethics Statement

All experimental procedures were conducted under national law and European Union directives on animal experiments and were approved by the animal welfare committee [dierexperimentencommissie (DEC)] of the University of Amsterdam.

## Author Contributions

SR and AK: conceptualization, writing—original draft, project administration. SR: formal analysis and visualization. AK: funding acquisition and supervision. SR, NS, and KYY: investigation. SR, AK, CL, PL, and ST: writing—review and editing. All authors contributed to the article and approved the submitted version.

## Conflict of Interest

The authors declare that the research was conducted in the absence of any commercial or financial relationships that could be construed as a potential conflict of interest.

## Publisher’s Note

All claims expressed in this article are solely those of the authors and do not necessarily represent those of their affiliated organizations, or those of the publisher, the editors and the reviewers. Any product that may be evaluated in this article, or claim that may be made by its manufacturer, is not guaranteed or endorsed by the publisher.
