# Supplementary material for: Modulation of the Hypothalamic Nutrient Sensing Pathways by Sex and Early-Life Stress
Source: Front Neurosci. 2021 Jul 23;15:695367. doi: 10.3389/fnins.2021.695367 (PMC8342927; doi:10.3389/fnins.2021.695367)
Supplement: Supplementary file 1 [file Table_1.DOCX]

Supplementary table 1. Primers for RT-PCR

| **Function** | **Gene** | **Forward primer** | **Reverse primer** |
| --- | --- | --- | --- |
| Fatty acid metabolism | *Acacb* | AGATGGCCGATCAGTACGTC | GGGGACCTAGGAAAGCAATC |
|  | *CPT1a* | GAGACTTCCAACGCATGACA | ATGGGTTGGGGTGATGTAGA |
|  | *CPT1c* | CGTGTCTGGAATGACTTTCTTGCT | TCTCCATCAGTCCTAGGGAAGG |
|  | *Fasn* | GCGCTCCTCGCTTGTCGTCT | TAGAGCCCAGCCTTCCATCTCCTG |
|  | *Ppara* | ATGCCAGTACTGCCGTTTTC | GGCCTTGACCTTGTTCATGT |
|  | *Ppard* | AACCCACGGTAAAGGCAGTC | CTTCCTCTTTCTCCTCTTCCCG |
|  | *Pparg* | GTCTCACAATGCCATCAGGTT | CAAATGCTTTGCCAGGGCTC |
|  | *Ppargc1a* | TGCTAGCGGTTCTCACAGAG | AGTGCTAAGACCGCTGCATT |
| Receptors | *Ghsr* | GATGCTTGCTGTGGTGGTGTT | GAAGAGGACAAAGGACACCAGG |
|  | *Insr* | GCCACTAATCCTTCTGTCCCC | AGGTAGTGTGTGATGTTGCCA |
| Neuropeptides | *Npy* | TCACCAGACAGAGATATGGCAA | AAGTCGGGAGAACAAGTTTCA |
|  | *Agrp* | AAGTCTGAATGGCCTCAAGAAGA | GACTCGTGCAGCCTTACACAG |
|  | *Pomc* | CGAGGCCTTTCCCCTAGAGT | CCAGGACTTGCTCCAAGCC |
| Reference genes | *Rpl13a* | CCCTCCACCCTATGACAAGA | CTGCCTGTTTCCGTAACCTC |
|  | *Rplp0* | GCTTCATTGTGGGAGCAGACA | CATGGTGTTCTTGCCCATCAG |
|  | *Tbp* | GTCATTTTCTCCGCAGTGCC | GCTGTTGTTCTGGTCCATGAT |
|  | *Tuba1a* | CCCTCGCCTTCTAACGCGTTGC | TGGTCTTGTCACTTGGCATCTGGC |
